# Structural insights into the architecture and assembly of eukaryotic flagella

**DOI:** 10.15698/mic2020.11.734

**Published:** 2020-09-21

**Authors:** Narcis-Adrian Petriman, Esben Lorentzen

**Affiliations:** 1 Department of Molecular Biology and Genetics, Aarhus University, Gustav Wieds Vej 10c, DK-8000 Aarhus C, Denmark.

**Keywords:** cilia, intraflagellar transport (IFT), microtubule doublets, BBSome

## Abstract

Cilia and flagella are slender projections found on most eukaryotic cells including unicellular organisms such as *Chlamydomonas, Trypanosoma* and *Tetrahymena,* where they serve motility and signaling functions. The cilium is a large molecular machine consisting of hundreds of different proteins that are trafficked into the organelle to organize a repetitive microtubule-based axoneme. Several recent studies took advantage of improved cryo-EM methodology to unravel the high-resolution structures of ciliary complexes. These include the recently reported purification and structure determination of axonemal doublet microtubules from the green algae *Chlamydomonas reinhardtii*, which allows for the modeling of more than 30 associated protein factors to provide deep molecular insight into the architecture and repetitive nature of doublet microtubules. In addition, we will review several recent contributions that dissect the structure and function of ciliary trafficking complexes that ferry structural and signaling components between the cell body and the cilium organelle.

## INTRODUCTION

Microbial cells often utilize cellular structures known as flagella to swim in aqueous environments. Flagellum-driven motility can occur in response to chemical stimuli (chemotaxis) or in response to a light source (phototaxis) and allows cells to swim towards beneficial environments. Bacterial flagella are surface appendages that serve as helical propellers often essential for the successful infection by pathogens [1]. Although different types of bacterial flagella exist, they have a common architecture based on a rotary ATPase motor that via Basal body and Hook structures links to and propels a long filament that typically extends from the cell body [2]. Archaeal microbes can also utilize flagella for motility, but these flagella are only superficially similar to the bacterial counterparts and have evolved independently to have different structures and assembly mechanisms [2].

Flagella are also found on eukaryotic microbes but are evolutionarily and structurally distinct from their prokaryotic counterparts. The motile eukaryotic flagellum (also known as a cilium) was discovered in 1675 by Antoine von Leeuwenhoek [3] and is conserved on a wide range of unicellular eukaryotic organisms such as the green alga *Chlamydomonas reinhardtii* [4], the parasite *Trypanosoma brucei* [5, 6], and the ciliates *Paramecium* and *Tetrahymena* [7]. Yeasts are important exceptions as they lack cilia, which were presumably lost during evolution. The flagellum on eukaryotic microorganisms, like that of prokaryotes, serves motility and sensory reception functions and allow cells to swim in response to external cues [8].

**Figure 1:**
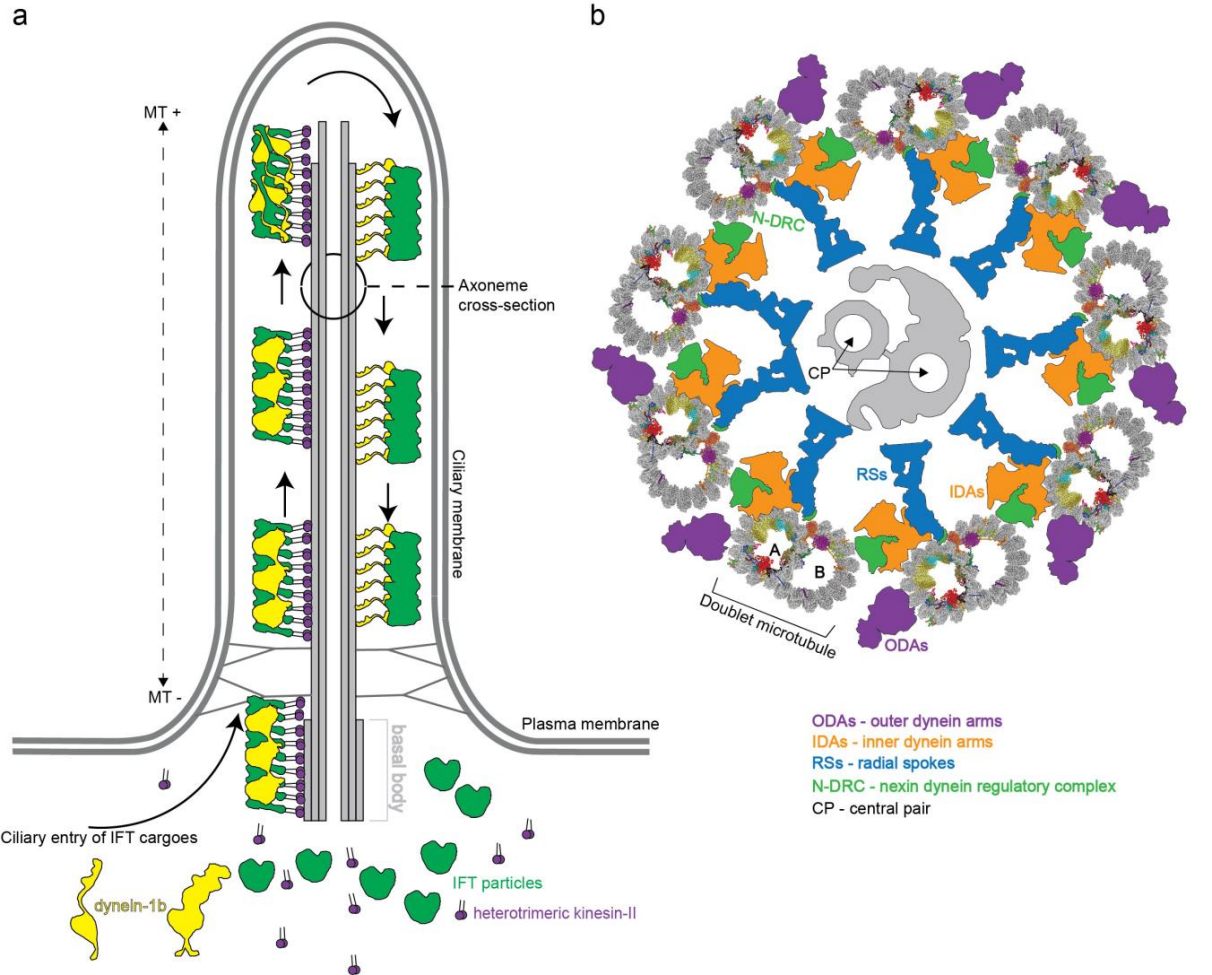
FIGURE 1: Schematic representation of a cilium from *Chlamydomonas reinhardtii*. **(a)** Architecture of a cilium with a simplified scheme of the bi-directional intraflagellar transport (IFT) system trafficking proteins between the cilium and the cell body. IFT complexes (green) assemble into train-like polymers powered by heterotrimeric kinesin-II (purple) in the anterograde direction (base->tip). Inactive dynein-1b (yellow) is loaded as a cargo onto anterograde IFT ‘trains'. Once assembled and loaded with ciliary cargo, these trains are driven across the transition zone and Y-links (depicted as grey connections spanning from the ciliary membrane to the axoneme at the ciliary base) to reach the ciliary tip. Upon arrival at the ciliary tip, the IFT ‘trains' are re-modelled, turnover products are picked up and moved back again to the ciliary base by retrograde IFT powered by the now activated dynein-1b motor. **(b)** Schematic representation of an axoneme cross section of a motile cilium, which depicts the nine peripheral doublet microtubules (DMTs; PDB entry: 6u42) that surround a central microtubule pair (CP, shown in grey). The DMTs are connected though the nexin dynein regulatory complex (N-DRC, shown in green). Complexes responsible for ciliary beating are the inner- and outer- dynein arms (IDAs and ODAs; orange and magenta, respectively). The central pair is connected to the nine DMTs by radial spokes (RS; blue).

In mammals, motile cilia power the sperm cells but are also present on the apical surface of epithelial cells where they generate external fluid flows [9]. This is the case for airway cilia that create a mucus flow to clear the lungs of dust particles and pathogens, and cilia in the fallopian tubes that create a fluid flow to move the egg cell from the ovaries to the uterus [10]. Additionally, many vertebrate cells express a single primary cilium that is non-motile but serves important functions in sensory reception and signalling [11]. Primary and motile cilia share a common architecture consisting of a basal body that templates the microtubule (MT)-based axoneme, and a ciliary membrane that is continuous with the plasma membrane but compositionally distinct [8] (**Figure 1a**). The basal body organizes the growth of nine MT triplets [12] that transition into doublets that constitute the structural backbone of the ciliary axoneme. A transition zone, formed by Y-shaped structures, bridges MTs and plasma membrane, separates the ciliary and cytosolic compartment and provides a selectivity barrier at the ciliary base [13–15]. In motile cilia, the nine outer doublet microtubules (DMTs) typically encircle two central MT singlets (**Figure 1b**). Motile cilia are also structurally different from primary cilia given that they harbour inner and outer dynein arms, which are macromolecular motor complexes that associate with DMTs and hydrolyse ATP to power the ciliary beat required for swimming [16–19] (**Figure 1b**). However, these macromolecular complexes are not present on all DMTs. One of the nine DMTs is lacking the outer dynein arms [20] while some inner dynein arms are missing in parts of the axoneme [21]. In addition, some of the dyneins have a preferred location at either the distal or the proximal end of the axoneme providing a longitudinal asymmetry [4, 22]. How DMTs maintain stability during ciliary beat has long been enigmatic given that cytoplasmic MTs easily break when exposed to similar forces [23–25]. It has however been evident from electron tomographic reconstructions of flagella *in situ* that the inner lumen of DMTs harbour large densities likely representing unknown protein factors [26–32]. Significant progress in understanding DMTs came with a recent publication where the authors made use of technical advances in cryo electron microscopy (cryo-EM) [33, 34] to obtain high-resolution single-particle reconstructions of DMTs, which allowed for the structural modelling of 33 MT inner proteins (MIPs) associated with the lumen of DMTs [35]. Important implications for DMT stability and periodicity gleaned from these structures will be highlighted in this review.

## ARCHITECTURE OF THE AXONEME

Proteomics studies reveal that as many as 500-1000 unique proteins participate in the construction of the motile eukaryotic flagellum [36, 37]. How all these factors associate to assemble ciliary axonemes has been a major topic of structural studies by EM since the early 1950. Initial studies of negatively stained axonemes from different species revealed the 9+2 arrangement [38–40] where the two central fibrils have a different size than that of the nine outer fibrils [40]. We now know that this difference in size originates from doublet vs singlet MTs. In the early 1960s it was indeed shown that these 9+2 fibril structures consist of MTs with similar properties to MTs found in the cytoplasm of cells [41]. Both cytoplasmic and axonemal MTs are built from a protofilament (PF) of α- and β-tubulin heterodimers, which provide a polarity for MT-based axoneme with (+) at the tip and (–) at the base (**Figure 1a**). MTs often contain 13 PFs although the number may vary [41]. Cytoplasmic MTs are dynamic structures that undergo cycles of polymerization and depolymerization of αβ-tubulin and are key players in cellular processes such as cell motility and cell division as well as intracellular transport [42].

The repetitive nature of 8.0 nm αβ-heterodimeric tubulins dictates a periodic arrangement of DMT-binding proteins. This axonemal periodicity is advantageous in structural studies as it allows for averaging of computationally extracted 48 nm or 96 nm repeating units imaged by cryo electron tomography (cryo-ET), which has been a powerful technique producing reconstructions at 20-40 angstroms (Å) resolutions [26–29, 34, 43–53]. These studies have determined the arrangement and conformation of several axonemal-associated protein complexes such as outer dynein arms (ODAs) and inner dynein arms (IDAs). ODAs and IDAs are ATP-hydrolysing motor complexes that facilitates ciliary beating through a controlled microtubule sliding motion [54] (**Figure 1b**). Another large axonemal macromolecular complex is the nexin-dynein regulatory complex (N-DRC) that bridges adjacent DMTs [28]. The N-DRC was recently shown to bind polyglutamylated tubulin via electrostatic interactions to regulate flagellar motility [55]. Additionally, the architecture of the T-shaped protein radial spoke (RS) complexes that connect DMTs to the central pair MTs has been resolved [47]. ODA, IDA, RS and N-DRC axonemal structures have been comprehensively reviewed by others [4, 56, 57] and will not be further covered here.

**Figure 2:**
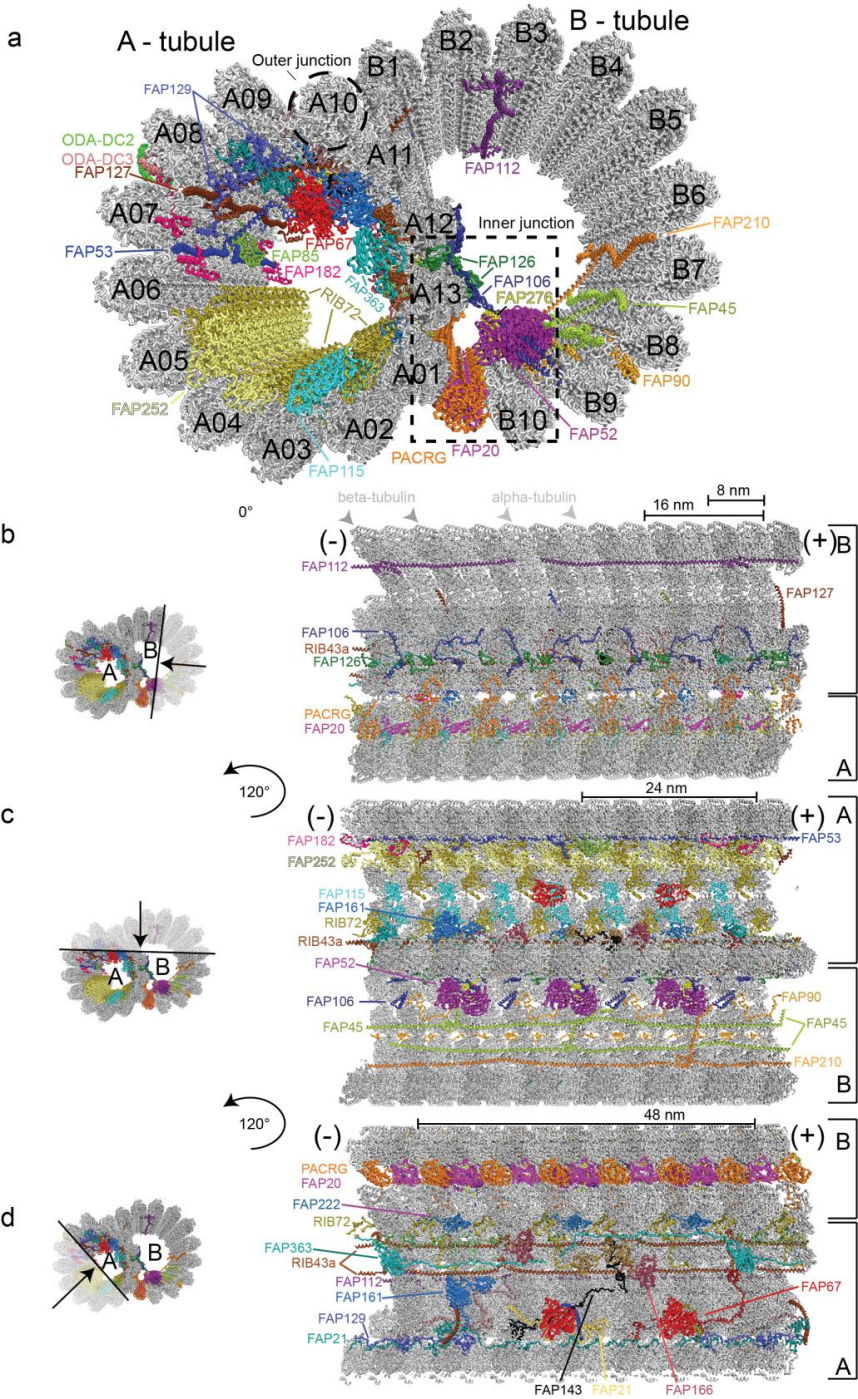
FIGURE 2: Structure of the 48nm ciliary doublet microtubule repeat (pdb entry: 6u42). **(a)** Cross section through a ciliary DMT. The outer dynein arms docking complexes 2 and 3 serve as docking sites for ODAs on the exterior of the DMTs and are labelled ODA-DC2 and ODA-DC3. Inner microtubule proteins (MIPs) are highlighted in different colours and labelled according to protein name. **(b-d)** Depiction of microtubule inner proteins, their interaction network and periodicity visualized within the confinements of the doublet microtubule from the minus (-) to the plus (+) end. **(b)** Lateral view of MIPs decorating the lumen of B-tubule as seen after a 10 nm deep slice facing the viewer was removed. The black arrow in the transparent region on the left representation indicates the region that has been removed for better clarity. The ODA-DC complex is left out as part of the removed section. **(c)** A lateral view displaying MIPs decorating both the A- and B-tubules. A 10 nm deep slice facing the viewer was removed as shown by the black arrow in the transparent region on the left representation. MIPs are visualized after rotation of the DMTs of 120° along the longitudinal axis relative to (b). **(d)** Visualisation of MIPs decorating the lumen of the A-tubule after removal of a 10 nm deep slice facing the viewer and rotation of the doublet microtubule with 120° along its longitudinal axis relative to (c). In this representation the alternating PCRG/FAP20 complex is observed from the exterior of the B-tubule.

DMTs are highly conserved structures built from an incomplete B-tubule fused onto a complete A-tubule composed of 13 tubulin PFs [24, 58] (**Figure 2**). Structural studies of axonemal MTs have a long history with the first three-dimensional models published as early as 1974 based on negatively stained electron micrographs, which clearly resolved the 8 nm repeat distance between αβ-tubulin heterodimers [59]. Analysis of these early negative stain EM pictures could however not clearly resolve if the B-tubule of DMTs consists of ten or eleven PFs [60]. However, with the improved resolution of cryo-ET reconstructions of *Chlamydomonas* and sea urchin sperm flagella it was clearly shown that the B-tubule consists of ten PFs [27, 30]. Extra density that could be mistaken for an 11^th^ PF instead corresponds to an inner junction protein complex, which attaches the B-tubule onto the A-tubule [30], and is composed of the FAP20/PACRG complex [61] (**Figure 2**). A subsequent study of DMTs used both cryo-ET and single particle cryo-EM to reach an improved resolution of 19 Å, which allowed for the exact assignment of the structurally similar α- and β-tubulin subunits within the DMTs based on EM data alone [51]. This study revealed that within the DMTs, the lattice pattern of tubulin isoforms is a B-lattice characterized by a left-handed helical arrangement of the PFs with a 0.92 nm stagger between α-α and β-β tubulins. Along the A-tubule, the 13 PFs are forming a discontinuity or a “seam” that lies between the PFs nine and ten [51, 59, 62].

## MOLECULAR STRUCTURE OF CILIARY DOUBLET MICROTUBULES AND ASSOCIATED PROTEIN FACTORS

Earlier studies have identified a class of MIPs associated with the lumen of *C. reinhardtii, T. thermophilia* and sea urchin sperm DMTs [27, 30, 63, 64]. The periodicity of MIPs with respect to the αβ-tubulin lattices has also been resolved [51] but the identity, structure and function of these MIPs remained enigmatic. A recent study pushed the boundaries of axonemal structural biology significantly by imaging extracted and purified *Chlamydomonas* axonemal DMTs by cryo-EM to obtain single-particle reconstructions of unprecedented resolution [35]. 3.6 Å resolution maps was obtained for the computationally extracted 96 nm DMT repeat whereas a somewhat better resolution of 3.4 Å was obtained for the 48 nm repeat. At this resolution, α- and β-tubulins as well as the respective nucleotide states (GDP vs GTP) could be distinguished in the cryo-EM maps [35]. These maps also allowed for the fold recognition and in most cases molecular modelling of 38 MT associated proteins of which 33 are MIPs associated with the lumen of DMTs [35]. Proteins seen to associate with the outer surface of DMTs include the ODA docking complex (ODA-DC), which is located between A07 and A08 PFs (A07-A08), has 24 nm periodicity and is required for the proper attachment of ODAs to the DMTs (**Figure 2a**). Furthermore, a 96 nm long coiled-coil segment was identified as Ccdc39/Ccdc40 [35] supporting the notion that this complex acts as a molecular ruler defining the 96 nm periodicity of the axoneme [65].

### Cryo-EM reconstruction of the 48 nm repeat allows structural modelling of 33 inner microtubule binding proteins

In the Ma *et al.* study, the 48 nm repeat reconstruction of DMTs displayed the highest resolution and allowed for the modelling the atomic structures of 33 MIPs (**Figure 2**). Molecular models were obtained by a combination of automatic fold recognition and chain tracing using the cryo-EM map [35]. To validate the correct assignment of proteins to their respective map densities, the structures of axonemal DMTs of mutants missing either the 72 kDa ribbon-associated protein RIB72 or the flagellar associated protein 166 (FAP166) were determined and the lack of density at the respective positions confirmed. Two isoforms of RIB72 were recently identified in *Tetrahymena* as MIPs associated with the lumen of the A-tubule and were shown to be important for ciliary assembly and motility [64]. Many more MIPs are found to associate with the lumen of the A-tubule than the B-tubule (**Figure 2**). An intricate network of protein-protein interactions connects different PFs with MIPs both within the respective tubules but also between A- and B-tubules [35] (**Figure 2**). These interactions likely account for the increased stability and longevity of axonemal DMTs compared to cytoplasmic MTs [23, 24]. Indeed, a recent study convincingly demonstrated using high-speed atomic force microscopy that FAP45 and FAP52, both proteins found in the lumen of DMTs, stabilize ciliary axonemes [25].

The 33 MIPs can be divided into different groups depending on their periodicity along the axoneme. MIPs with a periodicity of 8 nm such as FAP115, FAP252 and the C-terminal domains of RIB72 follow the 8 nm repeat distance of αβ-tubulin heterodimers and bind to protofilament A01-A05 (**Figure 2a** and **2c**). The PACRG/FAP20 complex that forms the inner junction between PF B10 of the B-tubule and A01 of the A-tubule also adheres to the 8 nm repeat although the ladder of PACRG/FAP20 is not completely continuous as one copy of PACRG is missing for every 96 nm repeat [30, 35]. *Chlamydomonas* mutants lacking FAP20 display split axonemes consistent with the role of PACRG/FAP20 in stabilizing the junction between A- and B-tubules [61]. The PACRG-FAP20 complex could be mistaken for a MT PF at lower resolution although this complex clearly has smaller overall dimensions than αβ-tubulin.

Interestingly, the N-terminal portion of RIB72 follows a 16 nm periodicity and interacts with other 16 nm repeats MIPs including FAP106, FAP126 and FAP52, which constitute a direct link between PFs of the A- and B-tubules (**Figure 2a** and **2b**). FAP52 makes extensive contacts to FAP276 that also follows 16 nm periodicity [66] (**Figure 2a**). Both FAP52 and FAP276 display an overall Y–shape and connect perpendicularly on the MTDs with the inner junction formed by the PACRG/FAP20 complex, the groove between microtubules B09 - B10 and FAP106 (**Figure 2a**, [35, 66]). Furthermore, both the N- and C-terminus of FAP276 contact the tubulin lattice [66], which may provide additional stability in gluing the A- and B-tubules together at the inner junction. Several MIPs extend from the internal lumen to the external surface of the DMTs where they may facilitate inside-outside communication and create new binding sites on the outside of DMTs. This is the case for the MIPs FAP85, FAP129 and FAP182 (48 nm periodicity) that bind in the cleft between PFs A07-A08 and contact ODA-DC on the exterior of DMTs (**Figure 2a**, [35]). FAP129 interacts with FAP127 on the inside and with ODA-DC on the exterior side, which may provide a molecular rationale for why FAP127 mutants show defects in ODA-DC assembly [67, 68]. The structure of DMTs presented by Ma *et al.* thus demonstrates that the distinction between inner and outer DMT-binding proteins is not so clear-cut and that MIPs may indeed protrude through ‘windows' between tubulin subunits to affect the exterior of DMTs [69].

Several MIPs polymerize along the axoneme via end-to-end interactions to establish periodicity. This is the case for the coiled-coil protein RIB43a, where two copies occupy the spaces between PFs A11-A12 and A12-A13 (**Figure 2d**). RIB43a self-associates along the DMTs through N-C termini interactions and the two copies of RIB43a are bridged by one copy of the protein FAP363 via a globular HSP70-like domain (**Figure 2a** and **2d**). FAP363 also self-assembles via N-C termini interactions. Many of the MIPs thus have the propensity for periodic assembly onto DMTs encoded by their amino acid sequence and thus do not rely solely on the repetitive nature of MTs to establish periodicity along the axoneme. These end-to-end MIPs also help define the periodicity of other MIPs by creating new binding sites at the lumen of DMTs [35]. Interestingly, these end-to-end MIPs are some of the first axonemal subunits to be expressed upon flagellum assembly [70], likely because of their important role in establishing the axonemal periodicity required for the attachment of other axonemal-associated proteins.

### The role of tubulin post-translational modifications as binding sites for MIPs

MTs undergo a wide range of post-translational modifications that constitute a tubulin code with important implications for MT function [71, 72]. These include (poly)glutamylation and (poly)glycylation added to α- and β-tubulin C-terminal tails found on the exterior of MTs. In the DMT structure presented by Ma *et al.* the C-terminal tubulin tails are mostly invisible in the cryo-EM map because of flexibility or proteolytic removal by the subtilisin enzyme used in DMT preparation. However, on PF A01, ten residues of the C-terminal tail of β-tubulin is visibly engaged in contacts with the PACRG/FAP20 complex and presumably strengthen this interaction, although it is not clear if post-translational modification plays a role here. Another long-studied MT modification is acetylation of the ε-group of the side-chain of lysine 40 (K40) in α-tubulin found on the luminal side of MTs [73, 74]. K40 acetylation is catalysed by α-tubulin acetyltransferase (aTAT1) [75–77] and occurs on polymerized MTs, which requires that αTAT1 gains access to the MT lumen either through openings at the MT ends or through tubulin lattice defects along the MTs [78–80]. It is a long-known fact that K40 acetylation correlates with a subset of long-lived MTs in cells but is has been a conundrum if K40 acetylation infers MT stability or if this modification is simply a mark that accumulates on ‘old' MTs [74, 81, 82]. It was shown that K40 acetylation does indeed provide mechanical stabilization of MTs [83]. Structural studies of acetylated MTs did, however, not provide a clear answer to the molecular basis given that the K40 loop region was disordered, although modelling did suggest that acetylation did reduce lateral contacts [84, 85]. Interestingly, in the structure of DMTs, the K40 loop is ordered on about 10% of the B-tubule and about 40% of the A-tubule where it participates in several interactions with various MIPs [35]. Acetylated K40-loops of α-tubulin thus serve as binding sites for MIPs and may indeed help organize the intricate network of MIPs bound at the lumen of DMTs. The structure thus suggests that K40 acetylation stabilize DMTs indirectly through the interactions with MIPs that in turn bridge PFs of both A- and B-tubules to create long-lived stable DMTs [35]. However, it is currently not known if K40 acetylation is required to recruit MIPs or if MIP-recruitment is a requirement for K40 acetylation.

## INTRAFLAGELLAR TRANSPORT ON DOUBLET MICROTUBULES

DMTs do not only serve as the structural backbone of flagella but also function as ‘tracks' for transport and delivery of protein cargoes. This intracellular trafficking system, known as intraflagellar transport (IFT), has evolved to ferry both structural and signalling components between the cell body and the cilium [86]. IFT was discovered in 1993 by Kozminski and Rosenbaum as a bi-directional transport process in the flagella of *Chlamydomonas* [87]. In *Chlamydomonas*, the molecular motor known as kinesin-II walks along DMTs towards the (+) end at the ciliary tip thus powering IFT in the anterograde direction [88–90]. The retrograde motor dynein1b or dynein-2 in mammals walks along DMTs towards the (–) of MTs bring IFT material to the base of the cilium [88, 91, 92]. IFT also relies on the large multi-subunit IFT particle for association with ciliary cargos such as tubulin [93–96] or ODAs [97–102] and utilize an octameric complex called the BBSome to couple the IFT system to various membrane associated signalling components for ciliary exit [103–105]. High-resolution structures are available for several IFT sub-complexes [106–110] and lower resolution structures of complete IFT trains were determined *in situ* by cryo-ET [111]. Furthermore, several recent structural cryo-EM studies of mammalian BBSome complexes were recently published [112–115]. The IFT and BBSome complexes were extensively reviewed previously and will not be further covered here [105, 116].

Given that kinesin and dynein motors both travel on DMTs, but in opposite directions, the question arises of why collisions between anterograde and retrograde IFT ‘trains' are not observed. At least two different models could allow for this: 1) Anterograde and retrograde trafficking could occur on different DMTs or 2) occur on different tubules of the same DMT, thus making the DMT a bi-directional double track for IFT [117]. An elegant study used correlative light and electron microscopy to show that hypothesis 2) is correct in *Chlamydomonas* where the kinesin-2 motors travel on B-tubules and the dynein-2 on A-tubules for retrograde transport [118]. Furthermore, in *Chlamydomonas,* all nine DMTs are actively used for IFT [118]. This situation is different in the unicellular parasite *Trypanosoma brucei* where only doublets 3-4 and 7-8 are utilized for IFT [117]. However, IFT was also observed to travel bi-directionally on each set of doublets in *Trypanosomes* suggesting that the double track mechanism may be evolutionarily conserved [117]. From the DMT structure, it is clear that IFT ‘trains' have limited physical space to access the MT PFs because of numerous other associated protein complexes (**Figure 1b**, [35]). This is the case for the A-tubule where only PFs A08-A10 are accessible to the IFT dynein motor. Kinesin-driven anterograde IFT ‘trains' have more space on the B-tubule but the fact that these IFT ‘trains' also contact the ciliary membrane suggests that they are restricted to PFs B01-04 [35, 118].

During anterograde IFT, inactive dynein is loaded onto IFT trains as a cargo and delivered to the ciliary tip [111, 119, 120]. Recent cryo-EM studies elucidated the structure of human dynein-2 at a resolution of 4.4 Å [120] and revealed a dual mechanism of inactivation during anterograde transport. Firstly, dynein-2 motor domains have an intrinsic propensity to stack against each other in an inactive dimeric conformation where the speed of dynein-2 on MTs is decreased to 140 nm/s [120]. Contrary, a dynein mutant that prevents dimerization walks along MTs with a velocity of 530 nm/s, which is in agreement with the speed of purified monomeric dynein-2 motor alone [119] and with the speed of retrograde IFT in mammalian cells [104]. Interestingly, the inactive conformation of dynein-2 dimers provided a good fit to the density in electron tomograms of IFT ‘trains' suggesting that this is indeed the state adopted by dynein-2 during anterograde IFT [111, 120]. Secondly, when the auto-inhibited dynein-2 dimer is loaded onto IFT trains, the MT-binding domains are oriented so that they point away from the MTs, which prevents dynein-2 in engaging the DMT tracks during anterograde IFT [111, 120].

Fluorescent labelling revealed that the anterograde IFT ‘trains' are fragmented at the ciliary tip, which suggest that a re-modeling event takes place [121–123]. Arrival of IFT ‘trains' at the ciliary tip allows for activation of dynein-2 [122, 124], engagement on the microtubule tracks for the retrograde IFT (**Figure 1a**) as well as kinesin-II detachment and diffusion or transport back to the cell body [121]. The re-configuration of dynein-2 at the ciliary tip is supported by cryo-EM studies that suggest an open conformation of dynein-2 motor domains [111] (**Figure 1a**). It is unclear how the inactive dynein-2 is converted at the tip to the active form that drives the retrograde IFT. Because no additional factors such as dynactin were found to modulated the activity of dynein-2 [125–127], it is possible that components of the IFT complex are responsible. There are three GTPases (IFT22, IFT27 and RabL2) embedded in the IFT ‘trains' travelling at different stages of anterograde IFT [106, 110, 128, 129]. Of these, IFT22 and IFT27 accompanies the IFT ‘trains' all the way to the tip where they may participate in remodelling of the IFT ‘trains'. Together with IFT25, IFT27 was shown to be required for the exit of certain GPCRs from cilia [130, 131] most likely indirectly by controlling ciliary trafficking of the BBSome. Interestingly, IFT27 seems to specifically sense ubiquitinated GPCRs and trigger their ciliary removal [132, 133]. Additionally, it was shown that the ubiquitination machinery is present in the flagella of *Chlamydomonas* [134] and that components of the IFT machinery interact with ubiquitinated proteins such as α-tubulin during cilia disassembly [135]. Furthermore, molecular genetics studies suggest that the membrane-associated IFT subunit IFT172 is also important for dynein-2 targeting or turnaround at the ciliary tip by an unknown mechanism that likely involves the *Chlamydomonas* microtubule end binding protein 1 [136–139]. Interestingly, IFT172 predominantly co-immunoprecipitates a version of the dynein-2 heavy chain that is somewhat larger in molecular mass suggesting that it could be modified, perhaps by ubiquitination [124]. Taken together, these data raise the possibility that ubiquitination plays important roles in IFT tip turnaround and retrograde transport. Another mechanism that can contribute to ciliary turnaround at the tip is phosphorylation. Specific kinases were discovered to localize at the tip of cilia both in *Chlamydomonas* and in mammalian cells targeting the kinesin-II motor and disrupting its interaction with IFT-B proteins [140, 141]. The comprehensive molecular mechanisms of dynein-2 activation and remodelling of anterograde to retrograde IFT ‘trains' remains to be elucidated.

## Abbreviations:

DMT: – doublet MT,
EM: – electron microscopy,
ET: – electron tomography,
IDA: – inner dynein arm,
IFT: – intraflagellar transport,
MIP: – MT inner protein,
MT: – microtubules,
N-DRC: – nexin-dynein regulatory complex,
ODA: – outer dynein arm.
PF: – protofilament,
RS: – radial spoke.

## References

[1] Chaban B, Hughes HV, Beeby M (2015). The flagellum in bacterial pathogens: For motility and a whole lot more.. Semin Cell Dev Biol.

[2] Jarrell KF, McBride MJ (2008). The surprisingly diverse ways that prokaryotes move.. Nat Rev Microbiol.

[3] Dobell C, Leeuwenhoek A van (1932). Antony van Leeuwenhoek and his “Little animals”; being some account of the father of protozoology and bacteriology and his multifarious discoveries in these disciplines.

[4] Ishikawa T (2017). Axoneme Structure from Motile Cilia.. Cold Spring Harb Perspect Biol.

[5] Langousis G, Hill KL (2014). Motility and more: the flagellum of Trypanosoma brucei.. Nat Rev Microbiol.

[6] Bertiaux E, Bastin P (2020). Dealing with several flagella in the same cell.. Cell Microbiol.

[7] Vincensini L, Blisnick T, Bastin P (2011). 1001 model organisms to study cilia and flagella.. Biol Cell.

[8] Ishikawa H, Marshall WF (2011). Ciliogenesis: building the cell's antenna.. Nat Rev Mol Cell Biol.

[9] Satir P, Christensen ST (2007). Overview of Structure and Function of Mammalian Cilia.. Annu Rev Physiol.

[10] Lyons RA, Saridogan E, Djahanbakhch O (2006). The reproductive significance of human Fallopian tube cilia.. Hum Reprod Update.

[11] Mourão A, Christensen ST, Lorentzen E (2016). The intraflagellar transport machinery in ciliary signaling.. Curr Opin Struct Biol.

[12] Li S, Fernandez J-J, Marshall WF, Agard DA (2012). Three-dimensional structure of basal body triplet revealed by electron cryo-tomography.. EMBO J.

[13] Gilula NB, Satir P (1972). THE CILIARY NECKLACE A Ciliary Membrane Specialization.. J Cell Biol..

[14] Gon?alves J, Pelletier and L (2017). The Ciliary Transition Zone: Finding the Pieces and Assembling the Gate.. Mol Cells.

[15] Greenan GA, Vale RD, Agard DA (2020). Electron cryotomography of intact motile cilia defines the basal body to axoneme transition.. J Cell Biol.

[16] Summers KE, Gibbons IR (1971). Adenosine Triphosphate-Induced Sliding of Tubules in Trypsin-Treated Flagella of Sea-Urchin Sperm.. Proc Natl Acad Sci U S A.

[17] Pfister KK, Witman GB (1984). Subfractionation of Chlamydomonas 18 S dynein into two unique subunits containing ATPase activity.. J Biol Chem.

[18] Kagami O, Kamiya R (1992). Translocation and rotation of microtubules caused by multiple species of Chlamydomonas inner-arm dynein.. J Cell Sci.

[19] Kamiya R, Jeon KW (2002). Functional diversity of axonemal dyneins as studied in Chlamydomonas mutants.. International Review of Cytology..

[20] Hoops HJ, Witman GB (1983). Outer doublet heterogeneity reveals structural polarity related to beat direction in Chlamydomonas flagella.. J Cell Biol.

[21] Bui KH, Yagi T, Yamamoto R, Kamiya R, Ishikawa T (2012). Polarity and asymmetry in the arrangement of dynein and related structures in the Chlamydomonas axoneme.. J Cell Biol.

[22] Ichikawa M, Bui KH (2018). Microtubule Inner Proteins: A Meshwork of Luminal Proteins Stabilizing the Doublet Microtubule.. BioEssays.

[23] Behnke O, Forer A (1967). Evidence For Four Classes of Microtubules in Individual Cells.. J Cell Sci.

[24] Witman GB, Carlson K, Berliner J, Rosenbaum JL (1972). CHLAMYDOMONAS FLAGELLA I. Isolation and Electrophoretic Analysis of Microtubules, Matrix, Membranes, and Mastigonemes.. J Cell Biol.

[25] Owa M, Uchihashi T, Yanagisawa H, Yamano T, Iguchi H, Fukuzawa H, Wakabayashi K, Ando T, Kikkawa M (2019). Inner lumen proteins stabilize doublet microtubules in cilia and flagella.. Nat Commun.

[26] Nicastro D, Schwartz C, Pierson J, Gaudette R, Porter ME, McIntosh JR (2006). The Molecular Architecture of Axonemes Revealed by Cryoelectron Tomography.. Science.

[27] Sui H, Downing KH (2006). Molecular architecture of axonemal microtubule doublets revealed by cryo-electron tomography.. Nature.

[28] Heuser T, Raytchev M, Krell J, Porter ME, Nicastro D (2009). The dynein regulatory complex is the nexin link and a major regulatory node in cilia and flagella.. J Cell Biol.

[29] Bui KH, Sakakibara H, Movassagh T, Oiwa K, Ishikawa T (2009). Asymmetry of inner dynein arms and inter-doublet links in Chlamydomonas flagella.. J Cell Biol.

[30] Nicastro D, Fu X, Heuser T, Tso A, Porter ME, Linck RW (2011). Cryo-electron tomography reveals conserved features of doublet microtubules in flagella.. Proc Natl Acad Sci U S A.

[31] Pigino G, Ishikawa T (2012). Axonemal radial spokes.. BioArchitecture..

[32] Ichikawa M, Liu D, Kastritis PL, Basu K, Hsu TC, Yang S, Bui KH (2017). Subnanometre-resolution structure of the doublet microtubule reveals new classes of microtubule-associated proteins.. Nat Commun.

[33] Kühlbrandt W (2014). The Resolution Revolution.. Science.

[34] Song K, Shang Z, Fu X, Lou X, Grigorieff N, Nicastro D (2020). In situ structure determination at nanometer resolution using TYGRESS.. Nat Methods.

[35] Ma M, Stoyanova M, Rademacher G, Dutcher SK, Brown A, Zhang R (2019). Structure of the Decorated Ciliary Doublet Microtubule.. Cell.

[36] Pazour GJ, Agrin N, Leszyk J, Witman GB (2005). Proteomic analysis of a eukaryotic cilium.. J Cell Biol.

[37] Blackburn K, Bustamante-Marin X, Yin W, Goshe MB, Ostrowski LE (2017). Quantitative Proteomic Analysis of Human Airway Cilia Identifies Previously Uncharacterized Proteins of High Abundance.. J Proteome Res.

[38] Fawcett DW, Porter KR (1954). A study of the fine structure of ciliated epithelia.. J Morphol.

[39] Sedar AW, Beams HW, Janney CD (1952). Electron Microscope Studies on the Ciliary Apparatus of the Gill Cells of Mya arenaria.. Proc Soc Exp Biol Med.

[40] Manton I, Clarke B (1952). An Electron Microscope Study of the Spermatozoid of Sphagnum.. J Exp Bot.

[41] Ledbetter MC, Porter KR (1964). Morphology of microtubules of plant cells.. Science.

[42] Goodson HV, Jonasson EM (2018). Microtubules and Microtubule-Associated Proteins.. Cold Spring Harb Perspect Biol.

[43] Lin J, Tritschler D, Song K, Barber CF, Cobb JS, Porter ME, Nicastro D (2011). Building Blocks of the Nexin-Dynein Regulatory Complex in Chlamydomonas Flagella.. J Biol Chem.

[44] Barber CF, Heuser T, Carbajal-González BI, Botchkarev VV, Nicastro D (2011). Three-dimensional structure of the radial spokes reveals heterogeneity and interactions with dyneins in Chlamydomonas flagella.. MBoC.

[45] Lin J, Heuser T, Carbajal-González BI, Song K, Nicastro D (2012). The structural heterogeneity of radial spokes in cilia and flagella is conserved.. Cytoskeleton.

[46] Heuser T, Dymek EE, Lin J, Smith EF, Nicastro D (2012). The CSC connects three major axonemal complexes involved in dynein regulation.. MBoC.

[47] Pigino G, Bui KH, Maheshwari A, Lupetti P, Diener D, Ishikawa T (2011). Cryoelectron tomography of radial spokes in cilia and flagella.. J Cell Biol.

[48] Oda T, Yanagisawa H, Kikkawa M (2014). Detailed structural and biochemical characterization of the nexin-dynein regulatory complex.. MBoC.

[49] Song K, Awata J, Tritschler D, Bower R, Witman GB, Porter ME, Nicastro D (2015). In Situ Localization of N and C Termini of Subunits of the Flagellar Nexin-Dynein Regulatory Complex (N-DRC) Using SNAP Tag and Cryo-electron Tomography.. J Biol Chem.

[50] Awata J, Song K, Lin J, King SM, Sanderson MJ, Nicastro D, Witman GB (2015). DRC3 connects the N-DRC to dynein g to regulate flagellar waveform.. MBo..

[51] Maheshwari A, Obbineni JM, Bui KH, Shibata K, Toyoshima YY, Ishikawa T (2015). α- and β-Tubulin Lattice of the Axonemal Microtubule Doublet and Binding Proteins Revealed by Single Particle Cryo-Electron Microscopy and Tomography.. Structure.

[52] Kubo T, Hou Y, Cochran DA, Witman GB, Oda T (2018). A microtubule-dynein tethering complex regulates the axonemal inner dynein f (I1).. Mol Biol Cell.

[53] Gui L, Song K, Tritschler D, Bower R, Yan S, Dai A, Augspurger K, Sakizadeh J, Grzemska M, Ni T, Porter ME, Nicastro D (2019). Scaffold subunits support associated subunit assembly in the Chlamydomonas ciliary nexin–dynein regulatory complex.. Proc Natl Acad Sci U S A.

[54] Lin J, Nicastro D (2018). Asymmetric distribution and spatial switching of dynein activity generates ciliary motility.. Science..

[55] Kubo T, Oda T (2017). Electrostatic interaction between polyglutamylated tubulin and the nexin-dynein regulatory complex regulates flagellar motility.. Mol Biol Cell.

[56] Mizuno N, Taschner M, Engel BD, Lorentzen E (2012). Structural Studies of Ciliary Components.. J Mol Biol.

[57] Osinka A, Poprzeczko M, Zielinska MM, Fabczak H, Joachimiak E, Wloga D (2019). Ciliary Proteins: Filling the Gaps. Recent Advances in Deciphering the Protein Composition of Motile Ciliary Complexes.. Cells.

[58] Warner FD, Satir P (1973). The Substructure of Ciliary Microtubules.. J Cell Sci.

[59] Amos LA, Klug A (1974). Arrangement of subunits in flagellar microtubules.. J Cell Sci.

[60] Linck RW, Stephens RE (2007). Functional protofilament numbering of ciliary, flagellar, and centriolar microtubules.. Cell Motil Cytoskeleton.

[61] Yanagisawa H, Mathis G, Oda T, Hirono M, Richey EA, Ishikawa H, Marshall WF, Kikkawa M, Qin H (2014). FAP20 is an inner junction protein of doublet microtubules essential for both the planar asymmetrical waveform and stability of flagella in Chlamydomonas.. MBoC.

[62] Kikkawa M, Ishikawa T, Nakata T, Wakabayashi T, Hirokawa N (1994). Direct visualization of the microtubule lattice seam both in vitro and in vivo.. J Cell Biol.

[63] Pigino G, Maheshwari A, Bui KH, Shingyoji C, Kamimura S, Ishikawa T (2012). Comparative structural analysis of eukaryotic flagella and cilia from Chlamydomonas, Tetrahymena, and sea urchins.. J Struct Biol.

[64] Stoddard D, Zhao Y, Bayless BA, Gui L, Louka P, Dave D, Suryawanshi S, Tomasi RF-X, Dupuis-Williams P, Baroud CN, Gaertig J, Winey M, Nicastro D (2018). Tetrahymena RIB72A and RIB72B are microtubule inner proteins in the ciliary doublet microtubules.. MBoC.

[65] Oda T, Yanagisawa H, Kamiya R, Kikkawa M (2014). A molecular ruler determines the repeat length in eukaryotic cilia and flagella.. Science.

[66] Khalifa AAZ, Ichikawa M, Dai D, Kubo S, Black CS, Peri K, McAlear TS, Veyron S, Yang SK, Vargas J, Bechstedt S, Trempe J-F, Bui KH The inner junction complex of the cilia is an interaction hub that involves tubulin post-translational modifications.. eLife.

[67] Ta-Shma A (2018). Homozygous loss-of-function mutations in MNS1 cause laterality defects and likely male infertility.. PLOS Genet.

[68] Zhou J, Yang F, Leu NA, Wang PJ (2012). MNS1 Is Essential for Spermiogenesis and Motile Ciliary Functions in Mice.. PLOS Genet.

[69] Ichikawa M, Khalifa AAZ, Kubo S, Dai D, Basu K, Maghrebi MAF, Vargas J, Bui KH (2019). Tubulin lattice in cilia is in a stressed form regulated by microtubule inner proteins.. Proc Natl Acad Sci U S A.

[70] Albee AJ, Kwan AL, Lin H, Granas D, Stormo GD, Dutcher SK (2013). Identification of Cilia Genes That Affect Cell-Cycle Progression Using Whole-Genome Transcriptome Analysis in Chlamydomonas reinhardtti.. G3.

[71] Gadadhar S, Bodakuntla S, Natarajan K, Janke C (2017). The tubulin code at a glance.. J Cell Sci.

[72] Wloga D, Joachimiak E, Fabczak H (2017). Tubulin Post-Translational Modifications and Microtubule Dynamics.. Int J Mol Sci.

[73] L'Hernault SW, Rosenbaum JL (1985). Chlamydomonas alpha-tubulin is posttranslationally modified by acetylation on the epsilon-amino group of a lysine.. Biochemistry.

[74] Piperno G, LeDizet M, Chang XJ (1987). Microtubules containing acetylated alpha-tubulin in mammalian cells in culture.. J Cell Biol.

[75] Shida T, Cueva JG, Xu Z, Goodman MB, Nachury MV (2010). The major α-tubulin K40 acetyltransferase αTAT1 promotes rapid ciliogenesis and efficient mechanosensation.. Proc Natl Acad Sci U S A.

[76] Akella JS, Wloga D, Kim J, Starostina NG, Lyons-Abbott S, Morrissette NS, Dougan ST, Kipreos ET, Gaertig J (2010). MEC-17 is an α-tubulin acetyltransferase.. Nature.

[77] Taschner M, Vetter M, Lorentzen E (2012). Atomic resolution structure of human α-tubulin acetyltransferase bound to acetyl-CoA.. Proc Natl Acad Sci U S A..

[78] Coombes C, Yamamoto A, McClellan M, Reid TA, Plooster M, Luxton GWG, Alper J, Howard J, Gardner MK (2016). Mechanism of microtubule lumen entry for the α-tubulin acetyltransferase enzyme αTAT1.. Proc Natl Acad Sci U S A.

[79] Ly N, Elkhatib N, Bresteau E, Piétrement O, Khaled M, Magiera MM, Janke C, Le Cam E, Rutenberg AD, Montagnac G (2016). αTAT1 controls longitudinal spreading of acetylation marks from open microtubules extremities.. Sci Rep.

[80] Szyk A, Deaconescu AM, Spector J, Goodman B, Valenstein ML, Ziolkowska NE, Kormendi V, Grigorieff N, Roll-Mecak A (2014). Molecular Basis for Age-Dependent Microtubule Acetylation by Tubulin Acetyltransferase.. Cell.

[81] Maruta H, Greer K, Rosenbaum JL (1986). The acetylation of alpha-tubulin and its relationship to the assembly and disassembly of microtubules.. J Cell Biol.

[82] Janke C, Montagnac G (2017). Causes and Consequences of Microtubule Acetylation.. Curr Biol.

[83] Xu Z, Schaedel L, Portran D, Aguilar A, Gaillard J, Marinkovich MP, Théry M, Nachury MV (2017). Microtubules acquire resistance from mechanical breakage through intralumenal acetylation.. Science.

[84] Eshun-Wilson L, Zhang R, Portran D, Nachury MV, Toso DB, Löhr T, Vendruscolo M, Bonomi M, Fraser JS, Nogales E (2019). Effects of α-tubulin acetylation on microtubule structure and stability.. Proc Natl Acad Sci U S A.

[85] Portran D, Schaedel L, Xu Z, Théry M, Nachury MV (2017). Tubulin acetylation protects long-lived microtubules against mechanical ageing.. Nat Cell Biol.

[86] Pedersen LB, Rosenbaum JL (2008). Chapter Two Intraflagellar Transport (IFT): Role in Ciliary Assembly, Resorption and Signalling. In: Current Topics in Developmental Biology..

[87] Kozminski KG, Johnson KA, Forscher P, Rosenbaum JL (1993). A motility in the eukaryotic flagellum unrelated to flagellar beating.. Proc Natl Acad Sci U S A.

[88] Cole DG, Diener DR, Himelblau AL, Beech PL, Fuster JC, Rosenbaum JL (1998). Chlamydomonas Kinesin-II–dependent Intraflagellar Transport (IFT): IFT Particles Contain Proteins Required for Ciliary Assembly in Caenorhabditis elegans Sensory Neurons.. J Cell Biol.

[89] Kozminski KG, Beech PL, Rosenbaum JL (1995). The Chlamydomonas kinesin-like protein FLA10 is involved in motility associated with the flagellar membrane.. J Cell Biol.

[90] Walther Z, Vashishtha M, Hall JL (1994). The Chlamydomonas FLA10 gene encodes a novel kinesin-homologous protein.. J Cell Biol.

[91] Pazour GJ, Dickert BL, Witman GB (1999). The DHC1b (DHC2) Isoform of Cytoplasmic Dynein Is Required for Flagellar Assembly.. J Cell Biol.

[92] Porter ME, Johnson KA (1983). The interaction of Tetrahymena 30S dynein with bovine brain microtubules.. J Submicrosc Cytol.

[93] Bhogaraju S, Cajanek L, Fort C, Blisnick T, Weber K, Taschner M, Mizuno N, Lamla S, Bastin P, Nigg EA, Lorentzen E (2013). Molecular Basis of Tubulin Transport Within the Cilium by IFT74 and IFT81.. Science.

[94] Kubo T, Brown JM, Bellve K, Craige B, Craft JM, Fogarty K, Lechtreck KF, Witman GB (2016). Together, the IFT81 and IFT74 N-termini form the main module for intraflagellar transport of tubulin.. J Cell Sci.

[95] Lechtreck KF (2015). IFT–Cargo Interactions and Protein Transport in Cilia.. Trends Biochem Sci.

[96] Scholey JM (2012). Kinesin-2 Motors Transport IFT-particles, Dyneins and Tubulin Subunits to the Tips of C. elegans Sensory Cilia: Relevance to Vision Research?. Vision Res.

[97] Hou Y, Witman GB (2017). The N-terminus of IFT46 mediates intraflagellar transport of outer arm dynein and its cargo-adaptor ODA16.. MBoC.

[98] Hou Y, Qin H, Follit JA, Pazour GJ, Rosenbaum JL, Witman GB (2007). Functional analysis of an individual IFT protein: IFT46 is required for transport of outer dynein arms into flagella.. J Cell Biol.

[99] Desai PB, Freshour JR, Mitchell DR (2015). Chlamydomonas Axonemal Dynein Assembly Locus ODA8 Encodes a Conserved Flagellar Protein Needed for Cytoplasmic Maturation of Outer Dynein Arm Complexes.. Cytoskeleton.

[100] Dai J, Barbieri F, Mitchell DR, Lechtreck KF (2018). In vivo analysis of outer arm dynein transport reveals cargo-specific intraflagellar transport properties.. MBoC.

[101] Taschner M, Mourão A, Awasthi M, Basquin J, Lorentzen E (2017). Structural basis of outer dynein arm intraflagellar transport by the transport adaptor protein ODA16 and the intraflagellar transport protein IFT46.. J Biol Chem.

[102] Wang J, Taschner M, Petriman NA, Andersen MB, Basquin J, Bhogaraju S, Vetter M, Wachter S, Lorentzen A, Lorentzen E (2020). Purification and crystal structure of human ODA16: Implications for ciliary import of outer dynein arms by the intraflagellar transport machinery.. Protein Science.

[103] Lechtreck K-F, Johnson EC, Sakai T, Cochran D, Ballif BA, Rush J, Pazour GJ, Ikebe M, Witman GB (2009). The Chlamydomonas reinhardtii BBSome is an IFT cargo required for export of specific signaling proteins from flagella.. J Cell Biol.

[104] Ye F, Nager AR, Nachury MV (2018). BBSome trains remove activated GPCRs from cilia by enabling passage through the transition zone.. J Cell Biol.

[105] Wingfield JL, Lechtreck K-F, Lorentzen E (2018). Trafficking of ciliary membrane proteins by the intraflagellar transport/BBSome machinery.. Essays Biochem.

[106] Bhogaraju S, Taschner M, Morawetz M, Basquin C, Lorentzen E (2011). Crystal structure of the intraflagellar transport complex 25/27.. EMBO J.

[107] Taschner M, Kotsis F, Braeuer P, Kuehn EW, Lorentzen E (2014). Crystal structures of IFT70/52 and IFT52/46 provide insight into intraflagellar transport B core complex assembly.. J Cell Biol.

[108] Taschner M, Weber K, Mourão A, Vetter M, Awasthi M, Stiegler M, Bhogaraju S, Lorentzen E (2016). Intraflagellar transport proteins 172, 80, 57, 54, 38, and 20 form a stable tubulin-binding IFT-B2 complex.. EMBO J.

[109] Taschner M, Lorentzen A, Mourão A, Collins T, Freke GM, Moulding D, Basquin J, Jenkins D, Lorentzen E (2018). Crystal structure of intraflagellar transport protein 80 reveals a homo-dimer required for ciliogenesis.. eLife.

[110] Wachter S, Jung J, Shafiq S, Basquin J, Fort C, Bastin P, Lorentzen E (2019). Binding of IFT22 to the intraflagellar transport complex is essential for flagellum assembly.. EMBO J.

[111] Jordan MA, Diener DR, Stepanek L, Pigino G (2018). The cryo-EM structure of intraflagellar transport trains reveals how dynein is inactivated to ensure unidirectional anterograde movement in cilia.. Nat Cell Biol.

[112] Chou H-T, Apelt L, Farrell DP, White SR, Woodsmith J, Svetlov V, Goldstein JS, Nager AR, Li Z, Muller J, Dollfus H, Nudler E, Stelzl U, DiMaio F, Nachury MV, Walz T (2019). The Molecular Architecture of Native BBSome Obtained by an Integrated Structural Approach.. Structure.

[113] Klink BU, Gatsogiannis C, Hofnagel O, Wittinghofer A, Raunser S (2020). Structure of the human BBSome core complex.. eLife.

[114] Singh SK, Gui M, Koh F, Yip MC, Brown A (2020). Structure and activation mechanism of the BBSome membrane protein trafficking complex.. eLife.

[115] Yang S, Bahl K, Chou H-T, Woodsmith J, Stelzl U, Walz T, Nachury MV (2020). Near-atomic structures of the BBSome reveal the basis for BBSome activation and binding to GPCR cargoes.. eLife.

[116] Taschner M, Lorentzen E (2016). The Intraflagellar Transport Machinery.. Cold Spring Harb Perspect Biol.

[117] Bertiaux E, Mallet A, Fort C, Blisnick T, Bonnefoy S, Jung J, Lemos M, Marco S, Vaughan S, Trépout S, Tinevez J-Y, Bastin P (2018). Bidirectional intraflagellar transport is restricted to two sets of microtubule doublets in the trypanosome flagellum.. J Cell Biol.

[118] Stepanek L, Pigino G (2016). Microtubule doublets are double-track railways for intraflagellar transport trains.. Science.

[119] Toropova K, Mladenov M, Roberts AJ (2017). Intraflagellar transport dynein is autoinhibited by trapping of its mechanical and track-binding elements.. Nat Struct Mol Biol.

[120] Toropova K, Zalyte R, Mukhopadhyay AG, Mladenov M, Carter AP, Roberts AJ (2019). Structure of the dynein-2 complex and its assembly with intraflagellar transport trains.. Nat Struct Mol Biol.

[121] Chien A, Shih SM, Bower R, Tritschler D, Porter ME, Yildiz A (2017). Dynamics of the IFT machinery at the ciliary tip.. eLife.

[122] Mijalkovic J, van Krugten J, Oswald F, Acar S, Peterman EJG (2018). Single-Molecule Turnarounds of Intraflagellar Transport at the C. elegans Ciliary Tip.. Cell Rep.

[123] Buisson J, Chenouard N, Lagache T, Blisnick T, Olivo-Marin J-C, Bastin P (2013). Intraflagellar transport proteins cycle between the flagellum and its base.. J Cell Sci.

[124] Pedersen LB, Geimer S, Rosenbaum JL (2006). Dissecting the molecular mechanisms of intraflagellar transport in chlamydomonas.. Curr Biol.

[125] Reck-Peterson SL, Redwine WB, Vale RD, Carter AP (2018). The cytoplasmic dynein transport machinery and its many cargoes.. Nat Rev Mol Cell Biol.

[126] Asante D, Stevenson NL, Stephens DJ (2014). Subunit composition of the human cytoplasmic dynein-2 complex.. J Cell Sci.

[127] Roberts AJ (2018). Emerging mechanisms of dynein transport in the cytoplasm versus the cilium.. Biochem Soc Trans.

[128] Kanie T, Abbott KL, Mooney NA, Plowey ED, Demeter J, Jackson PK (2017). The CEP19-RABL2 GTPase Complex Binds IFT-B to Initiate Intraflagellar Transport at the Ciliary Base.. Dev Cell.

[129] Nishijima Y, Hagiya Y, Kubo T, Takei R, Katoh Y, Nakayama K (2017). RABL2 interacts with the intraflagellar transport-B complex and CEP19 and participates in ciliary assembly.. MBoC.

[130] Eguether T, San Agustin JT, Keady BT, Jonassen JA, Liang Y, Francis R, Tobita K, Johnson CA, Abdelhamed ZA, Lo CW, Pazour GJ (2014). IFT27 Links the BBSome to IFT for Maintenance of the Ciliary Signaling Compartment.. Dev Cell.

[131] Keady BT, Samtani R, Tobita K, Tsuchya M, San Agustin JT, Follit JA, Jonassen JA, Subramanian R, Lo CW, Pazour GJ (2012). IFT25 Links the Signal-Dependent Movement of Hedgehog Components to Intraflagellar Transport.. Dev Cell.

[132] Desai PB, Stuck MW, Lv B, Pazour GJ (2020). Ubiquitin links smoothened to intraflagellar transport to regulate Hedgehog signaling.. J Cell Biol.

[133] Shinde SR, Nager AR, Nachury MV (2020). Lysine63-linked ubiquitin chains earmark GPCRs for BBSome-mediated removal from cilia.. bioRxiv.

[134] Huang K, Diener DR, Rosenbaum JL (2009). The ubiquitin conjugation system is involved in the disassembly of cilia and flagella.. J Cell Biol.

[135] Wang Q, Peng Z, Long H, Deng X, Huang K (2019). Polyubiquitylation of α-tubulin at K304 is required for flagellar disassembly in Chlamydomonas.. J Cell Sci.

[136] Pedersen LB, Miller MS, Geimer S, Leitch JM, Rosenbaum JL, Cole DG (2005). Chlamydomonas IFT172 Is Encoded by FLA11, Interacts with CrEB1, and Regulates IFT at the Flagellar Tip.. Curr Biol.

[137] Tsao C-C, Gorovsky MA (2008). Different Effects of Tetrahymena IFT172 Domains on Anterograde and Retrograde Intraflagellar Transport.. MBoC.

[138] Williamson SM, Silva DA, Richey E, Qin H (2012). Probing the role of IFT particle complex A and B in flagellar entry and exit of IFT-dynein in Chlamydomonas.. Protoplasma.

[139] Wang Q, Taschner M, Ganzinger KA, Kelley C, Villasenor A, Heymann M, Schwille P, Lorentzen E, Mizuno N (2018). Membrane association and remodeling by intraflagellar transport protein IFT172.. Nat Commun.

[140] Chaya T, Omori Y, Kuwahara R, Furukawa T (2014). ICK is essential for cell type-specific ciliogenesis and the regulation of ciliary transport.. EMBO J.

[141] Liang Y, Pang Y, Wu Q, Hu Z, Han X, Xu Y, Deng H, Pan J (2014). FLA8/KIF3B Phosphorylation Regulates Kinesin-II Interaction with IFT-B to Control IFT Entry and Turnaround.. Dev Cell.

[142] The PyMOL Molecular Graphics System, Version 2.3.0.

